# Chronic Hepatitis E Virus Infection during Lymphoplasmacytic Lymphoma and Ibrutinib Treatment

**DOI:** 10.3390/pathogens8030129

**Published:** 2019-08-22

**Authors:** Bernhard Schlevogt, Volker Kinast, Julia Reusch, Andrea Kerkhoff, Dimas Praditya, Daniel Todt, Hartmut H. Schmidt, Eike Steinmann, Patrick Behrendt

**Affiliations:** 1 Department of Medicine B for Gastroenterology and Hepatology, University Hospital Muenster, 48149 Münster, Germany; 2 Institute of Experimental Virology, TWINCORE Centre for Experimental and Clinical Infection Research, a Joint Venture between the Medical School Hannover (MHH) and the Helmholtz Centre for Infection Research (HZI), 30625 Hannover, Germany; 3 Department for Molecular and Medical Virology, Faculty of Medicine, Ruhr University Bochum, 44801 Bochum, Germany; 4 Department of Medicine A, Hematology, Oncology and Pneumology, University Hospital Muenster, 48149 Münster, Germany; 5 Department of Gastroenterology, Hepatology and Endocrinology, Hannover Medical School, 30625 Hannover, Germany; 6 German Centre for Infection Research (DZIF), Partner-Site Hannover-Braunschweig, 30625 Hannover, Germany

**Keywords:** hepatitis E virus, ibrutinib, Bruton’s tyrosine kinase, chronification, lymphoplasmacytic lymphoma, immunosuppression

## Abstract

Hepatitis E virus (HEV) is an increasingly recognised pathogen, affecting several hundred thousand individuals in western countries each year. Importantly, the majority of immunocompromised individuals are not able to clear HEV but develop a chronic course of infection. In the case of lymphoma, which is an inherent immunosuppressive disease per se, chemotherapy can even further exacerbate the immunosuppressive status. As the mechanism of HEV chronification is barely understood, it is important to gain knowledge about the influence of chemotherapeutic drugs on the HEV replication cycle to guide rational clinical management of HEV infection in such patients. In this case report, a 70 year old man was diagnosed with lymphoplasmacytic lymphoma. As we observed the occurrence of chronic HEV after treatment with the Bruton’s tyrosine kinase (BTK) inhibitor ibrutinib *in vivo*, we investigated the influence of BTK signaling and ibrutinib treatment in the HEV replication cycle *in vitro*. First, we detected an HEV-induced mobilisation of BTK in human liver cells during HEV replication. A moderate antiviral effect against HEV replicating isolates including genotypes 1 and 3 was observed, suggesting that ibrutinib did not support HEV replication in a direct manner. Combinatory treatments of ibrutinib with ribavirin indicated that ibrutinib did not influence the antiviral effect of ribavirin. Taken together, chemotherapy targeting cellular factors for the treatment of lymphomas may be a neglected risk factor for the chronification of HEV. For ibrutinib, despite the upregulation of its target BTK during HEV replication, we observed neither a proviral effect on HEV replication nor an influence on the antiviral effect of ribavirin, suggesting that the chronification of HEV may be favoured by its immunosuppressive effect.

## 1. Introduction

Hepatitis E virus (HEV) is increasingly recognised as an important pathogen, affecting several hundred thousand individuals in western countries each year [1]. Although HEV usually is a self-limiting disease, immunocompromised individuals are at risk to develop a chronic course of infection [2], with rapid progression to fibrosis, cirrhosis or even the development of liver failure [3]. The mechanism of HEV chronification is barely understood so far and treatment options in chronically infected patients are limited to the reduction of immunosuppression [2], pegylated interferon-α [4] and the off-label therapeutic ribavirin. Ribavirin showed efficacy in about 85% but is accompanied in part by severe side effects [5,6]. Chronic infections have also been reported in patients with underlying haematological malignancies [7]. Corresponding treatment often includes drugs inhibiting the host cell cycle. The knowledge about their influence on the viral replication cycle is of utmost importance to guide rational clinical management of HEV infection in such patients.

## 2. Material and Methods

### 2.1. Cell Culture

HepG2 cells were cultured on rat collagen-coated (SERVA Electrophoresis GmbH, Heidelberg, Germany) culture plates in Dulbecco’s Modified Eagle Medium (4.5 g/L glucose, Gibco, Thermo Fisher Scientific, Waltham, MA, USA) containing 10% fetal calf serum (FCS, Gibco, Thermo Fisher Scientific, Waltham, MA, USA), 2 mM L-glutamine (Gibco, Thermo Fisher Scientific, Waltham, MS, USA), 0.1 mM non-essential amino acid (Invitrogen), 10 U/mL penicillin (Gibco, Thermo Fisher Scientific, Waltham, MA, USA) and 10 μg/mL streptomycin (Gibco, Thermo Fisher Scientific, Waltham, MA, USA) at 37 °C. 

### 2.2. Compounds and Reagents

Ribavirin was purchased from Sigma Aldrich, St. Louis, MO, USA. Ibrutinib was purchased from MedChemExpress (Sollentuna, Sweden). All compounds were stored and diluted according to the manufacturer’s recommendations.

### 2.3. HEV Plasmids and In Vitro Transcription

Three plasmid constructs harbouring a subgenomic HEV sequence coupled to a Gaussia luciferase, Firefly luciferase or GFP reporter gene were used to generate HEV *in vitro* transcripts as previously described [8]. One of the constructs derives from the gt1 Sar55/S17 strain (based on clone pSK E2, GenBank accession no.AF444002), two derive from the Kernow-C1 p6 genome (gt3; GenBank accession no.JQ679013) and one derives from the G3-HEV83-2-27 genome (gt3; GenBank accession no.AB740232) and was a kind gift from the laboratory of Takaji Wakita. Capping of all constructs was performed using Ribo m^7^G CapAnalog (Promega, Madison, WI, USA). 

### 2.4. HEV Replication Assay

First, 5 × 10^6^ HepG2 cells in 400 μL Cytomix containing 2 mM ATP and 5 mM glutathione were mixed with 5 μg of HEV RNA. Electroporation was carried out with a Gene Pulser system (Bio-Rad, Munich, Germany). Afterwards, cells were cultured in DMEM on collagen-coated plates. Compounds were added for 48 h and viral replication was determined by measuring luciferase activity.

### 2.5. Luciferase Assay

Supernatant of cells (Gaussia luciferase) or suspension containing lysed cell (Firefly luciferase), were added to a 96-well white, flat-bottom microplate followed by the detection of luminescence using a microplate reader (CentroXS3 LB960, Berthold technologies, Bad Wildbad, Germany). Coelenterazine (Gaussia luciferase) or D-Luciferin (Firefly luciferase) was used as a substrate.

### 2.6. Viability Assay

Cell viability was determined by performing an MTT (3-(4,5-Dimethyl-2-thiazolyl)-2,5-diphenyl-2-H-tetrazolium bromide) assay (Sigma-Aldrich, Munich, Germany), according to the manufacturer’s suggestions.

### 2.7. Antibody-Based Microarray

Mobilisation of signalling molecules upon HEV replication was analysed by performing an antibody-based microarray (Kinex™ KAM-900P, Kinexus, Vancouver, BC, Canada). In brief, HepG2 cells were transfected with 5 µg subgenomic HEV-p6-GFP transcripts, 5 µg transferRNA (Sigma Aldrich, St. Louis, MO, USA) served as a control. Cells were harvested 12 and 48 h post electroporation, according to the manufacturer’s protocol, and shipped to Kinexus for microarray analysis. The whole data set will be published elsewhere (Kinast et al., manuscript in preparation).

### 2.8. Statistical Methods

For data analysis, GraphPad Prism 8 software was used.

### 2.9. Ethics

The study was approved by the ethics committee of the medical council of Westfalen-Lippe in Münster, Germany (record 2010-192-f-S), and it conforms to the ethical guidelines of the 1975 Declaration of Helsinki. The patient gave written informed consent to participate in this study.

## 3. Case

**Figure 1:**
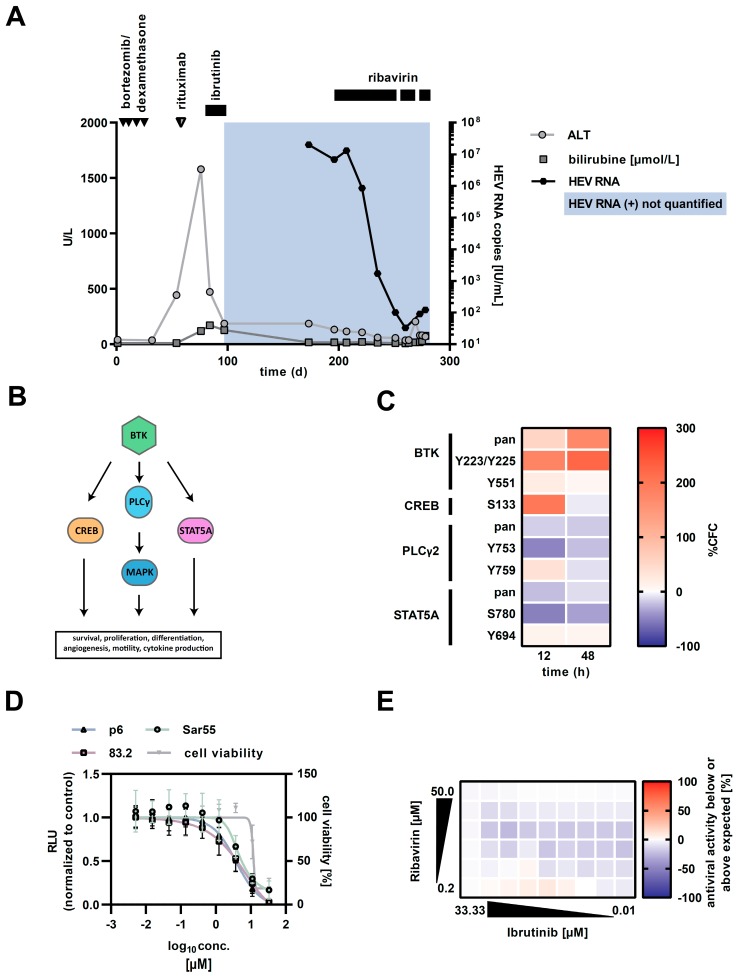
(**A**) Clinical course of index patient. Depicted are the courses of alanine amino-transferase (ALT; light gray line), bilirubine (medium gray line) and hepatitis E virus (HEV)-RNA in serum (black line) over the monitored time. Arrows and black horizontal bars indicate courses and period of application of medication, respectively. (**B**) Bruton’s tyrosine kinase. Depicted is a simplified scheme of Bruton’s tyrosine kinase (BTK), the downstream substrates of activated BTK and their associated signalling cascades. (**C**) Heatmap of altered expression levels and phosphorylation status of BTK and its downstream substrates in HEV-p6 replicating HepG2 cells, determined by an antibody microarray. (**D**) Effect of ibrutinib on HEV-p6 replication. Depicted is the dose-dependent inhibition of different HEV replicating isolates including genotypes 1 and 3 in HepG2 cells upon ibrutinib treatment for 48 h. Reduction in replication was determined via reporter luciferase read-out and normalised to the untreated control. Cell viability was monitored via MTT assay. Depicted are the mean values of three independent experiments. (**E**) Combinatory effect of ibrutinib and ribavirin on HEV-p6 replication. Heatmap indicates the potential differences between the actual experimental effects and the theoretical additive effects at various concentrations of the two compounds 48 h post electroporation. Depicted are the values of one representative experiment.

A 70 year old man was diagnosed with lymphoplasmacytic lymphoma. IgM was markedly elevated (717 mg/dL, normal: 40–230 mg/dL) and bone marrow infiltration was 40%. A typical MYD88-mutation was identified (c.794T > C). Throughout the entire course of the disease, the patient suffered from severe, progressive pancytopenia requiring constant transfusion of platelets and red blood cells. As initial treatment, the patient received four courses of bortezomib and dexamethasone (Figure 1A). However, pancytopenia did not improve. Follow-up bone marrow biopsies showed neither haematological reconstitution nor progressive or refractory lymphoma. As critical cytopenia persisted, the patient received a single infusion of rituximab without any improvement of bone marrow function. Concomitantly, the patient developed acute hepatitis E (genotype 3c) with peak alanine amino-transferase (ALT) at 1579 U/L (normal: 10–50 U/L). Apart from zoonotic transmission, HEV could have also been transmitted by repeated blood transfusions. Other viral infections causing hepatitis were excluded. Abdominal ultrasound showed no hepatic abnormality. Due to persistent cytopenia, treatment was escalated with the Bruton’s tyrosine kinase (BTK) inhibitor ibrutinib over 3 weeks, again with no effects on pancytopenia (Figure 1A). ALT levels initially declined, but then remained elevated at greater 100 U/L. Stimulation of bone marrow with granulocyte macrophage colony-stimulating factor (GM-CSF) was not successful. As HEV-RNA levels in blood (20,000,000 IU/mL) and faeces were positive for more than 3 months, chronic hepatitis E was diagnosed. CD19-positive B-cells were massively diminished in peripheral blood. As viral infections can cause pancytopenia, treatment with ribavirin was initiated despite concerns due to poor bone marrow function. Therefore, the dosage of ribavirin was slowly increased up to 1000 mg daily. Within 2 months of therapy, HEV-RNA decreased to 33 IU/mL followed by the normalisation of transaminases. Unfortunately, treatment had to be paused because of a severe exanthema associated with ribavirin. Despite the improvement of liver function, there was no recovery of pancytopenia, arguing against HEV-associated pancytopenia. After stopping ribavirin, the viral load increased again which was followed by an increase in transaminases and bilirubin (Figure 1A). The patient was able to restart ribavirin but died a few weeks later due to pulmonal mycosis caused by severe prolonged pancytopenia.

We observed the occurrence of chronic hepatitis E in the patient after ibrutinib treatment, concurrent with observations in French patients [9,10]. Due to the B-cell signalling inhibitory potential of ibrutinib, infections with hepatotropic viruses could be favoured, as has already been suggested for hepatitis B virus [11]. Therefore, we investigated the influence of BTK signalling and ibrutinib treatment in the HEV replication cycle *in vitro* in the hepatoma cell line HepG2. Although HepG2 cells may not always reflect the *in vivo* situation, they are a well-characterised, suitable and robust model to study liver-related diseases. First, we analysed the modulation of BTK during HEV replication (Figure 1B). We identified both an upregulation and phosphorylation of BTK as well as a phosphorylation of CREB (cAMP responsive element binding protein 1) in HEV replicating cells compared with mock transfected cells (Figure 1C), indicating an HEV-induced mobilisation of BTK in human liver cells. Next, we investigated the effect of an HEV infection by ibrutinib treatment on different HEV replicating isolates including genotypes 1 and 3. An antiviral effect against all tested HEV replicons upon treatment with 3.33 µM ibrutinib was observed without cytotoxicity (Figure 1D), suggesting that ibrutinib did not support HEV replication in a direct manner but rather had antiviral activity. Combinatory treatments of ibrutinib with ribavirin indicated that ibrutinib did not influence the antiviral effect of ribavirin (Figure 1E). Therefore, the clinical course of the patient cannot be explained by a direct effect of the drug but might be caused by the concomitant medication (e.g., rituximab), the influence of ibrutinib on cellular immunity or due to the underlying aplasia.

## 4. Conclusions

Lymphomas are inherent immunosuppressive diseases and chemotherapy can further exacerbate the immunosuppressive status of lymphoma patients. Chemotherapy targeting cellular factors for the treatment of lymphomas may be a neglected risk factor for the chronification of HEV. Therefore, for reasonable clinical decision-making, it is important to gain further knowledge about the influence of applied drugs on the HEV replication cycle. For ibrutinib, despite the upregulation of its target BTK during HEV replication, we observed neither a proviral effect on HEV replication nor an influence on the antiviral effect of ribavirin. Therefore, a possible proviral influence of this drug on HEV infection *in vivo*—as suggested by our case—may be favoured by its immunosuppressive effect.
